# THE IMPACT OF TECHNOLOGY USE ON CUSTODIAL GRANDPARENTS’ DEPRESSION

**DOI:** 10.1093/geroni/igac059.3100

**Published:** 2022-12-20

**Authors:** Sarah Pace, Yanfeng Xu, Theresa Harrison

**Affiliations:** University of South Carolina, Columbia, South Carolina, United States; University of South Carolina, Columbia, South Carolina, United States; University of South Carolina, Columbia, South Carolina, United States

## Abstract

During COVID-19, custodial grandparents experienced symptoms of depression due to social isolation, and technology use may reduce depression. This study examines the association between level of comfort of using technology and depression among custodial grandparents. Cross-sectional survey data (N = 287) were collected via multiple sources, including state agencies, local non-profit organizations serving kinship families, foster parent associations, schools, and Qualtrics Panels between March 2021 and March 2022. The average age of grandparents was 55 years. Over half were female (71.43%), had some college education (68.75%), and were White (55.12%). Depression was measured using the CES-D 10 scale. This variable was dummy coded using 8 as the cutoff score (Andresen et al., 1994). The level of comfort using technology was measured by combining four survey questions. Grandparent age, race, gender, marital status, home ownership, geographic area, disability, employment, education, physical health, telehealth, and telemental health were controlled. The logistic regression model revealed that those who had a higher comfort level with technology had significantly lower odds of having depression (OR = .543, p = < .001). Those with physical health had significantly lower odds of having depression (OR = .727, p = .044). Grandparents who needed telehealth (OR = 2.81, p =.005) and telemental health services (OR = 2.93, p =.003) had significantly higher odds of having depression. This research implies that custodial grandparents’ use of technology, particularly their comfort levels with technology may reduce depression among grandparents. This will inform practice and policy for those who work with custodial grandparents.

